# The relationship between number of pregnancies and serum 25-hydroxyvitamin D levels in women with a prior pregnancy: a cross - sectional analysis, machine learning - based prediction model, and SHAP - assisted feature importance evaluation

**DOI:** 10.3389/fendo.2025.1589002

**Published:** 2025-09-22

**Authors:** Ziyi Wu, Li Wei, Haichuan Zhang

**Affiliations:** ^1^ Department of Orthopedics, Second Hospital of Harbin Medical University, Harbin, China; ^2^ Chengdu Medical College, Chengdu, China

**Keywords:** gravidity, serum 25 (OH) D, vitamin D insufficiency, machine learning algorithms, predictive model

## Abstract

**Background:**

The primary aim of this study is to explore the association between gravidity and serum 25-hydroxyvitamin D [25(OH)D] levels in women, as existing research rarely addresses gravidity’s cumulative impact on maternal vitamin D status. Secondarily, it seeks to develop and evaluate a machine learning model for predicting vitamin D insufficiency (serum 25(OH)D < 50 nmol/L) using reproductive data (including gravidity) and biochemical indicators, with contribution analysis in the model further validating this relationship, thereby translating the findings into a clinically useful tool.

**Methods:**

The study included 8,003 parous women from the NHANES survey conducted between 2011 and 2018, excluding those with missing data on vitamin D or gravidity. For the primary objective, we employed covariate-adjusted linear regression analyses to examine the relationship between gravidity and serum 25(OH)D levels. Three hierarchical models were constructed: Model 1 (unadjusted); Model 2, adjusted for age and race/ethnicity; and Model 3, adjusted for all potential confounders (including body mass index, blood urea nitrogen, glycated hemoglobin, and diabetes status). For the secondary objective of model development, multiple regression analysis and six machine learning algorithms (including XGBoost and Random Forest) were employed. These algorithms are well-suited to handle mixed-type biomedical data (e.g., continuous biochemical indices and categorical reproductive factors), aligning with the characteristics of the dataset in this study. The dataset was subsequently split into a training set and a validation set at a 70:30 ratio.

**Results:**

The study found that each additional pregnancy was associated with a 0.6 nmol/L decrease in 25(OH)D concentration (P<0.001). For the secondary objective of predictive modeling, the XGBoost algorithm showed better performance in clinically predicting vitamin D levels, with an area under the receiver operating characteristic curve (AUC) of 0.73, which was superior to multiple regression analysis and the other five machine learning algorithms (including Random Forest, Logistic Regression, Support Vector Machine, Decision Tree, and Naive Bayes) and demonstrated greater efficacy in identifying low serum vitamin D levels. Key features contributing significantly to the model included age, body mass index (BMI), and blood urea nitrogen.

**Conclusion:**

In women with a prior pregnancy, an independent inverse association was observed between gravidity and vitamin D status and the XGBoost algorithm demonstrated superior performance in clinically predicting vitamin D levels using common blood test results which may facilitate timely detection and intervention for low serum vitamin D.

## 1 Introduction

25-hydroxyvitamin D [25(OH)D], a fat-soluble sterol metabolite, is the main form of vitamin D in circulation and acts as its principal storage form. 25(OH)D plays an important role in regulating calcium and phosphate balance in the body, mainly by increasing the absorption of these minerals in the intestines—a function important for maintaining skeletal health (1). Low serum levels of 25-hydroxyvitamin D [25(OH)D] have been known to be a common risk factor related to many diseases, including skeletal disorders, cardiovascular diseases, hypertension, autoimmune diseases, obesity, diabetes mellitus, chronic kidney disease, and depressive disorders (2–8). Approximately 30%-50% of the global population has severe 25(OH)D deficiency (serum levels < 10 ng/mL), and this condition is widespread even regions near the equator (9).

Existing research has placed emphasis on the association between vitamin D and pregnancy. Investigations show that low vitamin D status is strongly related to the development of gestational diabetes mellitus (GDM) (10). Low 25(OH)D levels in early pregnancy are associated with a higher incidence of preterm birth and may lead to poor long-term developmental outcomes in offspring (11). Research has looked at differences in gene expression among Black women, comparing those with preterm vs. term births, as well as those with different 25(OH)D levels and concentrations of vitamin D-binding protein (VDBP). Outcomes indicated that women with preterm births had 47 upregulated genes and 16 downregulated genes compared with those who had term births. Among women with low serum 25(OH)D, 361 genes were downregulated and 61 were upregulated compared with those who were vitamin D-sufficient. Additionally, women with low VDBP levels had 44 upregulated genes and 295 downregulated genes. Interestingly, many neutrophil-expressed genes were downregulated in both the low serum vitamin D group and the preterm birth group. These observations imply that vitamin D status, such as deficiency, could be used as an important clinical biomarker influencing the expression of genes associated with preterm birth (12).

Gravidity, defined as the total number of pregnancies, is known to raise the risk of several conditions, including atrial fibrillation (13), gynecological cancers (14), and gestational diabetes mellitus (15). Multiple pregnancies can disrupt a woman’s endocrine balance. However, there is currently insufficient research on the relationship between the number of pregnancies and women’s vitamin D status. Investigating this link might help explain how pregnancy count affects vitamin D concentrations and allow more specific nutritional advice to be provided to women with multiple pregnancies. In doing so, it might reduce the number of pregnancy complications in this population caused by low vitamin D.

The primary objective of this study is to examine the relationship between the number of pregnancies and serum 25-hydroxyvitamin D [25(OH)D] levels among American women, using data from the NHANES database. As a secondary objective, it aims to build a predictive model using machine learning techniques that combines pregnancy count and other basic biochemical indices to evaluate the risk of low serum vitamin D in women with a pregnancy-related history. As a rapidly developing discipline, machine learning has been widely applied in healthcare, manufacturing, education, and several other fields to support evidence-based decision-making (16). It utilizes large-scale data to construct robust predictive models that forecast the characteristics and risks of study populations (17). Machine learning-based screening tools can assess vitamin D insufficiency in study participants by processing large volumes of health data, with a stronger ability to handle complex variable relationships than traditional statistical methods—this provides methodological justification for the secondary objective of developing the predictive model.

## 2 Information and methodologies

### 2.1 Information and sample

This cross-sectional study analyzed data from the 2011–2012, 2013–2014, 2015–2016, and 2017–2018 cycles of the National Health and Nutrition Examination Survey (NHANES), which comprises 4 merged datasets. The Centers for Disease Control and Prevention (CDC) employs a complex multi-stage sampling method to assess the health and nutritional status of the population every two years. For the present study, these 4 consecutive NHANES cycles provided access to laboratory measurements of blood trace elements and comprehensive reproductive health questionnaires.

**Figure 1:**
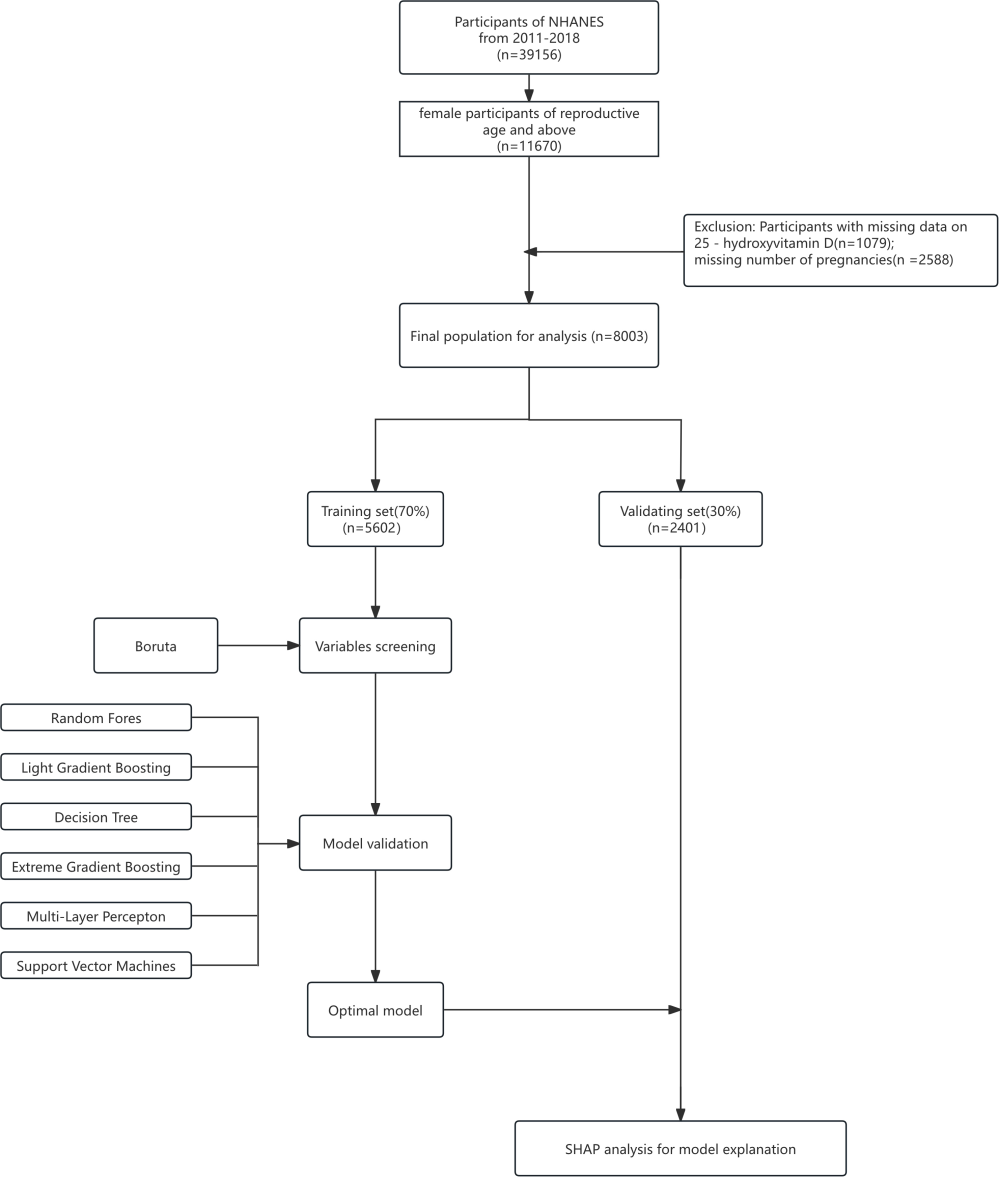
Training flow chart.

This study was conducted in compliance with the STROBE guidelines established by the EQUATOR Network. A total of 39,156 participants were included in the NHANES 2011–2018 surveys(reference **Figure 1**), with the number of participants per cycle as follows: 9,756 in 2011–2012, 10,175 in 2013–2014, 9,971 in 2015–2016, and 9,254 in 2017–2018. All participants in NHANES provided written informed consent, and all data collection procedures were approved by the Research Ethics Review Board of the National Center for Health Statistics (NCHS).

The original datasets and detailed documentation are publicly available via the NHANES official website (https://wwwn.cdc.gov/nchs/nhanes/Default.aspx). The eligibility criteria for this study were defined as follows: (1) female participants in the NHANES 2011–2018 cycles; (2) availability of complete data on serum 25-hydroxyvitamin D [25(OH)D] concentrations and number of pregnancies (gravidity); (3) Women with one or more pregnancies (4) women aged 20 years and older (including postmenopausal women). Currently pregnant participants were retained as they form a natural subgroup of the target population, and their exclusion would compromise the sample’s representativeness and external validity by distorting population structure (e.g., omitting pregnancy-related vitamin D metabolic traits in reproductive-aged women). Retention, with statistical adjustment for their potential impact, ensures more authentic reflection of association patterns between vitamin D levels and pregnancy-related factors. Additionally, excluding this subgroup (if constituting ≥5% of the sample) could reduce sample size, weaken statistical power, and destabilize subgroup analyses; statistical adjustment instead maximizes data retention, ensuring precision and reliability for core analyses (e.g., gravidity-vitamin D association). The selection process for the study population is presented in **Figure 1**. Following the screening strategy described by Wu et al., the included data comprised laboratory measurements of serum 25(OH)D levels, age at first birth (AFLB), number of pregnancies, and number of live births. Among the 39,156 total participants, 31,153 were excluded due to missing information on serum vitamin D levels or number of pregnancies.

### 2.2 Measurement of serum 25(OH)D concentration

In NHANES, serum 25-hydroxyvitamin D [25(OH)D] concentrations were quantified using the DiaSorin radioimmunoassay (RIA) kit (Stillwater, MN) during the 2001–2006 cycles, while the standardized liquid chromatography-tandem mass spectrometry (LC-MS/MS) method was employed from 2007 to 2014. To ensure comparability across the dataset, regression-based conversion was applied to translate RIA-derived 25(OH)D values into equivalents aligned with the standardized LC-MS/MS method, thereby correcting for assay drift. Consistent with recommendations from the CDC, the LC-MS/MS-derived data were used for the present investigation.

### 2.3 Reproductive health questionnaire

This study was conducted in compliance with the STROBE guidelines established by the EQUATOR Network. A total of 39,156 participants were included in the NHANES 2011–2018 surveys, with the number of participants per cycle as follows: 9,756 in 2011–2012, 10,175 in 2013–2014, 9,971 in 2015–2016, and 9,254 in 2017–2018 (reference **Figure 1**).

Reproductive health questionnaire procedures at the Mobile Examination Center (MEC) included items such as menstrual cycle, pregnancy history, and use of hormone replacement therapy. Trained interviewers at the MEC used the Computer-Assisted Personal Interview (CAPI) system to conduct the survey. Age at first birth (AFLB) and other relevant variables were determined based on participants’ responses. The number of pregnancies was derived from responses to the question: “How many times have you (or the study participant) been pregnant? Please include all pregnancies, such as current ones, live births, miscarriages, stillbirths, ectopic pregnancies, or abortions.” The number of live births was obtained from responses to: “How many of your/her deliveries resulted in live births?” For cases of multiple births in a single delivery, this was recorded as 1 live birth. Additionally, data on age at first pregnancy were collected.

For the purpose of this analysis, the gravidity variable was constructed using these self-reported data on the total number of pregnancies, encompassing all outcomes specified in the questionnaire. This variable was treated as a continuous metric in statistical models to quantify the cumulative effect of multiple pregnancies.

### 2.4 Covariate assessments

During in-home interviews, participants’ demographic data, including age and race/ethnicity, were been in survey via standardized questionnaires. BMI was calculated from measurements in NHANES 2011–2018 as weight (in kilograms) divided by the square of height (in meters). Overweight was defined as a BMI ≥25.0 kg/m². Diabetes was defined as HbA1c ≥6.5% or a “yes” response to the question: “Has a health professional ever told you that you have diabetes, excluding during pregnancy?”

Demographic variables such as age and ethnicity were obtained through standardized home interviews. Using NHANES 2011–2018 measurements, BMI was calculated as weight (kg) divided by height squared (m²). A BMI ≥25.0 kg/m² was classified as overweight. Diabetes was diagnosed based on either a glycated hemoglobin (HbA1c) level ≥6.5% or an affirmative answer the exam: “Has a doctor ever told you that you have diabetes, not including gestational diabetes?”

Race/ethnicity was categorized as Mexican American, other Hispanic, non-Hispanic White, non-Hispanic Black, or other racial groups. Educational level was stratified into three tiers: less than high school, high school or equivalent, and college or higher.

As part of NHANES laboratory testing from 2011–2018, serum samples were collected following rigorous protocols. Baseline measurements included glycated hemoglobin (HbA1c, %), direct high-density lipoprotein (HDL) cholesterol, alanine aminotransferase (ALT, U/L), creatinine (μmol/L), blood urea nitrogen (BUN), and aspartate aminotransferase (AST, U/L).

### 2.5 Statistical analysis

NHANES data are designed to be analyzed using complex survey weights to ensure representativeness of the target population, but no survey weights were applied in this study. This is because the primary focus of the study is to explore the associative patterns between gravidity and serum 25(OH)D levels and to develop machine learning-based predictive models, rather than to generate nationally representative estimates of population parameters (such as the prevalence of vitamin D insufficiency). Existing research indicates that when the focus is on relationships between variables rather than population inference, excluding weights has minimal impact on the direction and strength of associations and can more clearly reflect the underlying relational patterns within the sample. Additionally, the machine learning algorithms employed in this study (e.g., XGBoost) face methodological challenges in integrating complex survey weights, which may increase the complexity of model construction and hinder the capture of variable interaction effects. To prioritize the fitting accuracy of the predictive model for the associative features within the sample, weights were excluded from the analysis. Information analysis in this research was obtained accordance with the recommendations of the U.S. Centers for Disease Control and Prevention (CDC) (18).For the purposes of this study, the 8,003 participants were divided into two groups according to the criteria of <50.00 nmol/L and ≥50.00 nmol/L. The chi-square (χ²) test was applied to analyze categorical variables, while a linear regression analysis model was used to determine the associations between the 25(OH)D groups (categorized based on insufficiency cutoff of <50 nmol/L) and gravidity (total number of pregnancies reported) as the outcome variable. This linear regression model adjusted for potential confounding factors such as age, race/ethnicity, educational level, and body mass index to quantify the independent relationship between vitamin D status and pregnancy count. Among these participants, 2,383 had serum 25(OH)D levels <50.00 nmol/L, and 5,620 had levels ≥50.00 nmol/L. Continuous variables were reported as mean ± standard deviation, and categorical variables as percentages.

To investigate the relationship between the number of pregnancies and serum 25-hydroxyvitamin D [25(OH)D] levels, we performed covariate-adjusted analyses and multivariate linear regression. Three models were included in the study: Model 1 (unadjusted); Model 2, adjusted for age and race; and Model 3, adjusted for all covariates listed in **Table 1**. The results are presented in **Table 2**.

**Table 1:** Description of training set participants’ characteristics, shows the participant characteristics in the training set.

Variable	25(OH)D<50	>= 50?	Standardize diff.	P-value	P-value*
	N=2383	N=5620			
Weight (kg)	82.9 ± 23.7	75.3 ± 19.7	0.3 (0.3, 0.4)	<0.001	<0.001
Age	47.0 ± 15.6	54.7 ± 16.4	0.5 (0.4, 0.5)	<0.001	<0.001
Alt (U/L)	20.4 ± 15.7	20.8 ± 13.5	0.0 (-0.0, 0.1)	0.241	<0.001
Creatinine	68.4 ± 43.7	70.6 ± 30.4	0.1 (0.0, 0.1)	0.010	<0.001
Uric Acid (μmol/L)	289.3 ± 81.2	292.9 ± 78.5	0.0 (-0.0, 0.1)	0.060	0.048
ALP (U/L)	75.2 ± 27.0	71.7 ± 26.7	0.1 (0.1, 0.2)	<0.001	<0.001
BUN (mmol/L)	4.3 ± 2.1	5.1 ± 2.2	0.4 (0.3, 0.4)	<0.001	<0.001
AST (U/L)	22.8 ± 21.9	23.1 ± 12.0	0.0 (-0.0, 0.1)	0.489	<0.001
TG (mg/dL)	137.8 ± 155.1	143.1 ± 94.4	0.0 (-0.0, 0.1)	0.061	<0.001
Chol (mg/dL)	189.8 ± 41.6	198.5 ± 41.4	0.2 (0.2, 0.3)	<0.001	<0.001
Direct HDL - C (mg/dL)	54.6 ± 15.9	58.8 ± 16.4	0.3 (0.2, 0.3)	<0.001	<0.001
BMI (kg/m²)	32.1 ± 8.4	29.5 ± 7.2	0.3 (0.3, 0.4)	<0.001	<0.001
HbA1c (%)	5.9 ± 1.4	5.8 ± 1.0	0.1 (0.1, 0.2)	<0.001	0.109
Vit D total	35.9 ± 9.3	83.6 ± 27.4	2.3 (2.3, 2.4)	<0.001	<0.001
No. of Pregnancies	3.5 ± 2.0	3.4 ± 2.0	0.0 (-0.0, 0.1)	0.068	0.096
AFLB	24.5 ± 56.7	24.2 ± 41.4	0.0 (-0.0, 0.1)	0.740	<0.001
Race/Hisp			0.7 (0.7, 0.8)	<0.001	–
Mexican American	464 (19.5%)	694 (12.3%)			
Non-Hispanic Black	247 (10.4%)	663 (11.8%)			
Non-Hispanic White	442 (18.5%)	2574 (45.8%)			
Other Hispanic	935 (39.2%)	915 (16.3%)			
Other Race	295 (12.4%)	774 (13.8%)			
Diabetes			0.0 (-0.0, 0.1)	0.550	–
Yes	333 (14.0%)	824 (14.7%)			
No	1983 (83.2%)	4615 (82.1%)			
Borderline	65 (2.7%)	178 (3.2%)			

After adjusting for pre-specified confounding variables, the probability that the observed current research results (or, more extreme results) are entirely due to random errors is indicated by the P-value*.

**Table 2:** Univariate and multivariate results by linear regression.

Variable	Model 1		Model 2		Model 3	
	β (95%CI)	P value	β (95%CI)	P value	β (95%CI)	P value
Number of pregnancies						
continuous	-0.50 (-0.85, -0.16)	0.0046	-0.76 (-1.09, -0.43)	<0.0001	-0.57 (-0.90, -0.25)	0.0005
Number of pregnancies						
0 (n = 1062)	0	0	0	0	0	0
1 (n = 925)	0.83 (-1.58, 3.24)	0.4978	-2.88 (-5.05, -0.71)	0.0093	-2.57 (-4.68, -0.45)	0.0173
2 (n = 925)	0.77 (-1.65, 3.19)	0.5332	-2.72 (-4.92, -0.53)	0.0149	-2.42 (-4.55, -0.28)	0.0268
3 (n = 925)	-1.00 (-3.23, 1.22)	0.3781	-4.42 (-6.48, -2.36)	<0.0001	-3.56 (-5.57, -1.55)	0.0005

Univariate and multivariate results by linear regression analysis was performed to explore the linear relationship between the number of continuous variable pregnancies and serum 25(OH)D levels. The study included three models: Model 1 without variable adjustment; Model 2 adjusted for age and race; and Model 3 adjusted for all covariates listed in **Table 1**: Weight (kg), Age, Alt (U/L), Creatinine, Uric Acid (μmol/L), ALP (U/L), BUN (mmol/L), AST (U/L), TG (mg/dL), Chol (mg/dL), Direct HDL - C (mg/dL), BMI (kg/m²), HbA1c (%), No. of Pregnancies, AFLB, Race, Diabetes.

### 2.6 Model development, assessment, and verification

Data from four cycles of the NHANES database (2011–2018) were included in the analysis. These data were randomly split into a training set and a validation set at a 70:30 ratio. The extracted variables were used as features for the machine learning (ML) analysis. Six ML algorithms were employed to build classification models, specifically: Random Forest (RF), Extreme Gradient Boosting (XGBoost), Light Gradient Boosting Machine (LightGBM), Decision Tree, Multilayer Perceptron (MLP), and Support Vector Machine (SVM).

To ensure the stability and repeatability of model performance, 10-fold cross-validation was repeated 10 times. Receiver Operating Characteristic (ROC) curves were constructed to evaluate the discriminative ability of the models, and the Area Under the Curve (AUC) of the ROC curve was calculated. The area was chosen to be the primary indicator to assess how well models could distinguish between vitamin D-deficient and sufficient individuals.

For a more detailed assessment of the models’ predictive efficacy, this study also reported additional metrics: Accuracy (ACC), Positive Predictive Value (PPV), Negative Predictive Value (NPV), Sensitivity (SEN), Specificity (SPE), F1-score, and Matthews Correlation Coefficient (MCC). Generally, the closer these statistical values are to 1, the better the predictive performance of the model.

The Kappa value to assess the consistency between the predicted results and the actual outcomes. Kappa values range from -1 to +1, with values closer to 1 indicating greater consistency. A Kappa score bigger than 0.75 reflects excellent function.

The Brier Score, which combines model discrimination and calibration, was used to evaluate the overall performance of the models. A Brier score approaching 0 indicates a higher degree of consistency between predicted values and actual observations. In this study, Decision Curve Analysis (DCA) was used to assess the practical value of the models in clinical decision-making.

The optimal deep learning prediction model was chosen using the AUC statistic as the main criterion, supplemented by a comprehensive evaluation of other statistical indicators. We used SHAP (Shapley Additive Explanations) values in our analysis. In our SHAP analysis, each data point shows how strongly a feature (e.g., number of pregnancies or BMI) affects the model’s prediction of vitamin D insufficiency for a participant. Notably, age has the largest average impact, a pattern specific to our dataset focused on reproductive health. Additionally, measures were taken to enhance the usability of this top-performing model in real-world settings. All information analyses, model building, and testing procedures in this study were conducted using R (version 4.1.3) and Python (version 3.13.1).

## 3 Results

### 3.1 Baseline characteristics of study participants

Regarding age characteristics, the mean age was approximately 52.44 years (SD = 16.56), with a range from 20 to 80 years. 25% of participants were 39 years old or younger, 50% were 53 or younger, and 75% were 65 or younger, indicating a somewhat middle - aged - centered distribution.

In terms of educational attainment, it was diverse. Those with “college or associate degrees” were the most numerous, accounting for 33.35% (n = 2669), followed by “high school graduates/high school equivalency (GED) or equivalent” at 22.17% (n = 1774) and “post - secondary or higher education” at 21.85% (n = 1749). The proportions of those with “9 - 11th grade (including 12th grade without a diploma)” and “below 9th grade” were 12.85% (n = 1028) and 9.71% (n = 777) respectively. Only 0.06% (n = 5) reported “don’t know” and 0.01% (n = 1) refused to disclose their educational information.

For racial/ethnic characteristics, the distribution was also diverse. Non-Hispanic Whites were the largest group, making up 37.69% (n = 3016), followed by Non-Hispanic Blacks at 23.12% (n = 1850), Mexican Americans at 14.47% (n = 1158), “Other Races - including multi-racial” at 13.36% (n = 1069), and Other Hispanics at 11.37% (n = 910). Summary statistics for key variables across this diverse population were as follows: serum 25(OH)D levels had a mean of 68.3 nmol/L (median: 63.1 nmol/L), with the prevalence of vitamin D deficiency (defined as <50 nmol/L) at 29.4%. For parity, the mean number of live births was 2.2 (median: 2), while gravidity (total pregnancies) had a mean of 2.7 (median: 2) and ranged from 1 to 12. These characteristics, including both demographic diversity and clinical metrics, provide important background information for further research based on this dataset.

### 3.2 Association between the number of pregnancies and 25(OH)D

**Table 2** shows the association between the number of pregnancies and total 25(OH)D levels. All models indicated a negative correlation between the number of pregnancies and total 25(OH)D. After adjusting for all confounding variables, each additional pregnancy was significantly associated with a 0.6 nmol/L decrease in total 25(OH)D (Model 3: b = -0.6, 95% CI: -0.9, -0.2).

The total number of pregnancies was divided into quartiles, and this association remained statistically significant. Compared with participants in the lowest quartile of pregnancy count, those in the highest quartile had a 3.6 nmol/L decrease in total 25(OH)D per additional pregnancy (b = -3.6, 95% CI: -5.6, -1.5; trend p < 0.001).

### 3.3 Model performance

**Figure 2:**
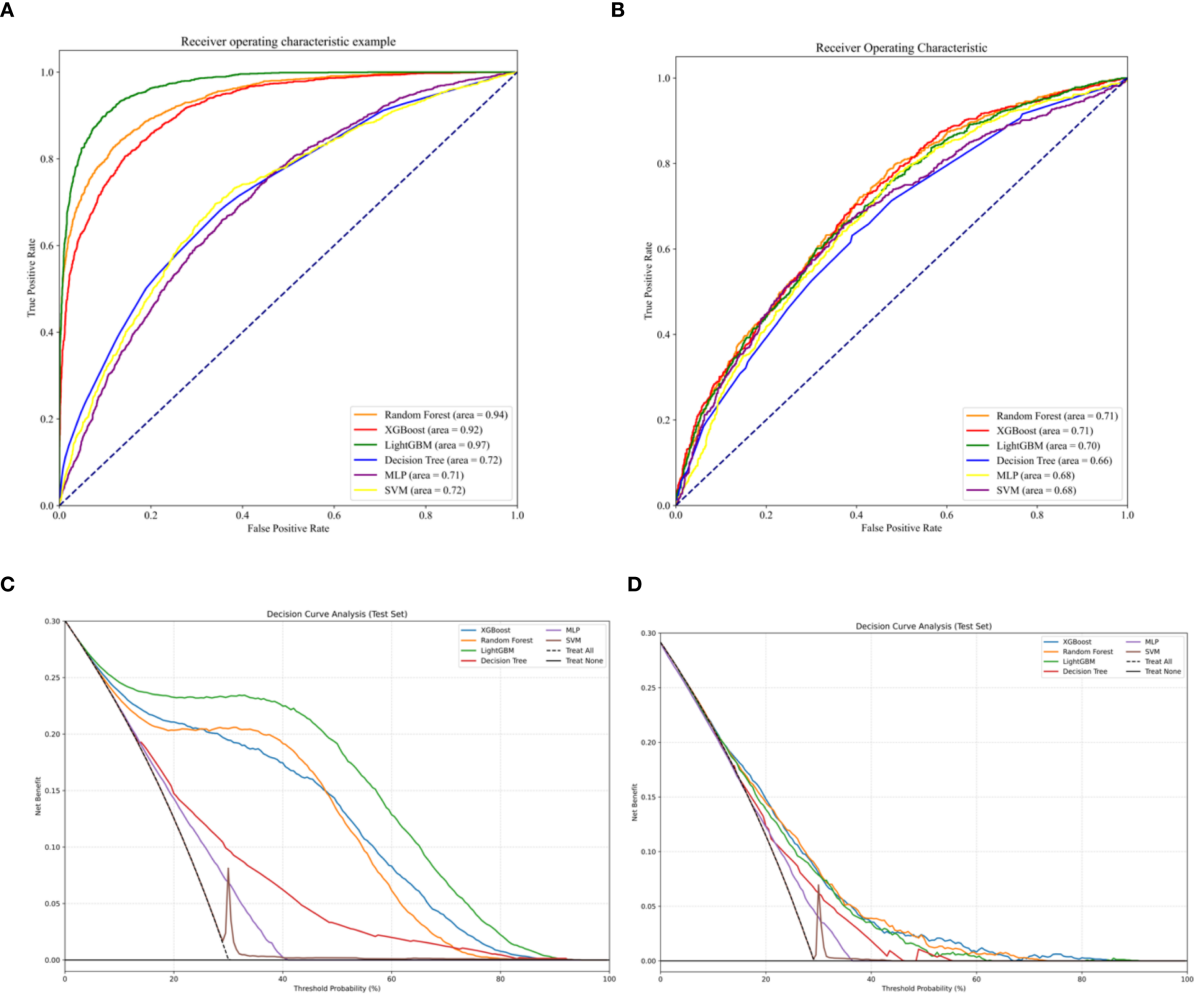
**(A)** ROC in the training set. **(B)** ROC in the validation set. **(C)** DCA curves in the training set. **(D)** DCA curves in the validation set.

**Figure 3:**
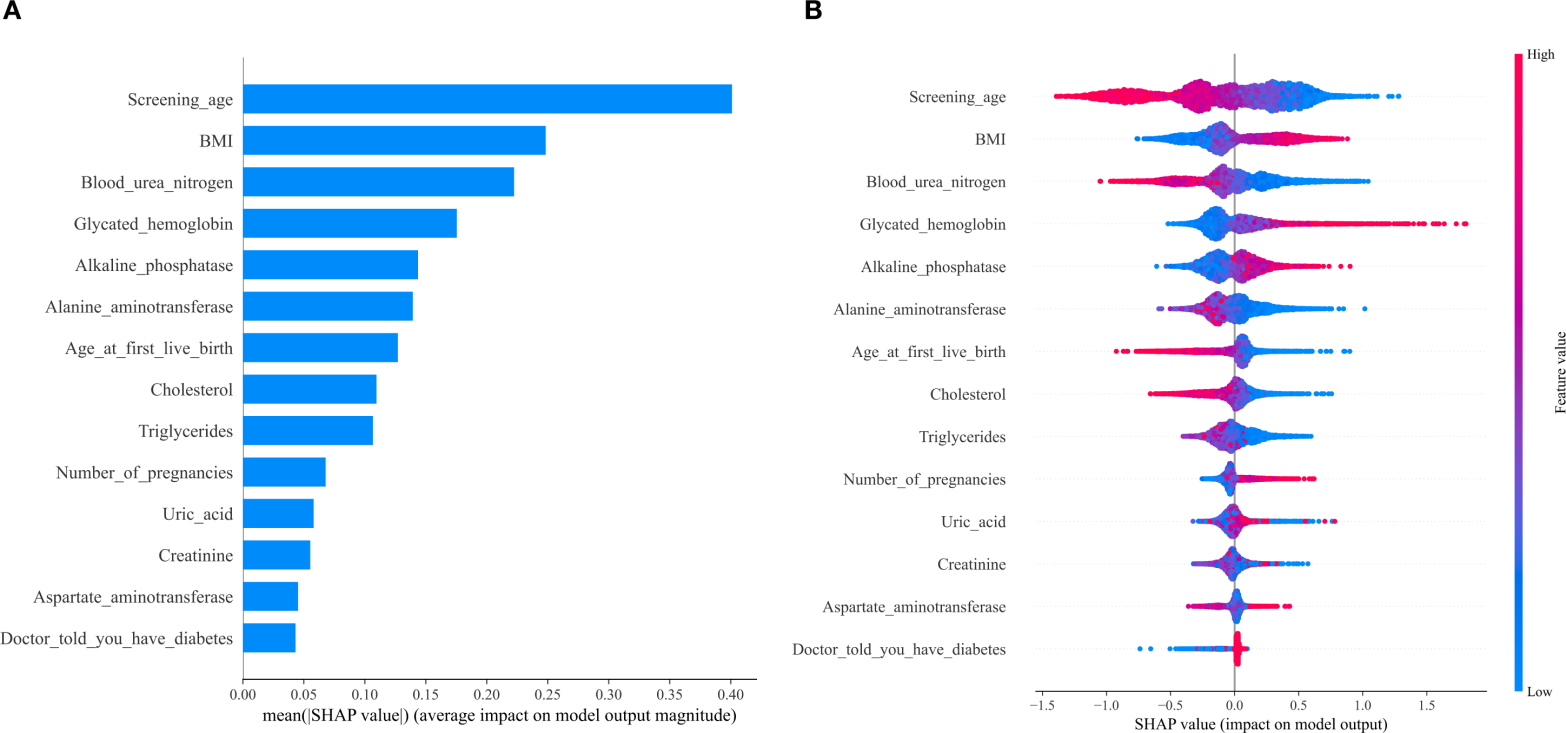
SHAP value ranked among the variables in the model. **(A)** SHAP value ranking of the variables in the model. **(B)** SHAP honeycomb diagram of the XGBoost model.

Area Under the Curve (AUC) values were calculated based on Receiver Operating Characteristic (ROC) curves (see **Figures 2A, B**). During training, LightGBM achieved the highest AUC (0.97), followed by Random Forest (RF, 0.94) and XGBoost (0.92); Decision Tree, Multilayer Perceptron (MLP), and Support Vector Machine (SVM) lagged behind with AUCs of 0.72, 0.71, and 0.72, respectively. Among all models, the one constructed using the XGBoost algorithm demonstrated the best predictive performance in both the training and validation sets, as determined by the AUC metric. **Figure 3** illustrates the performance of the various machine learning models on both sets.

**Figure 4:**
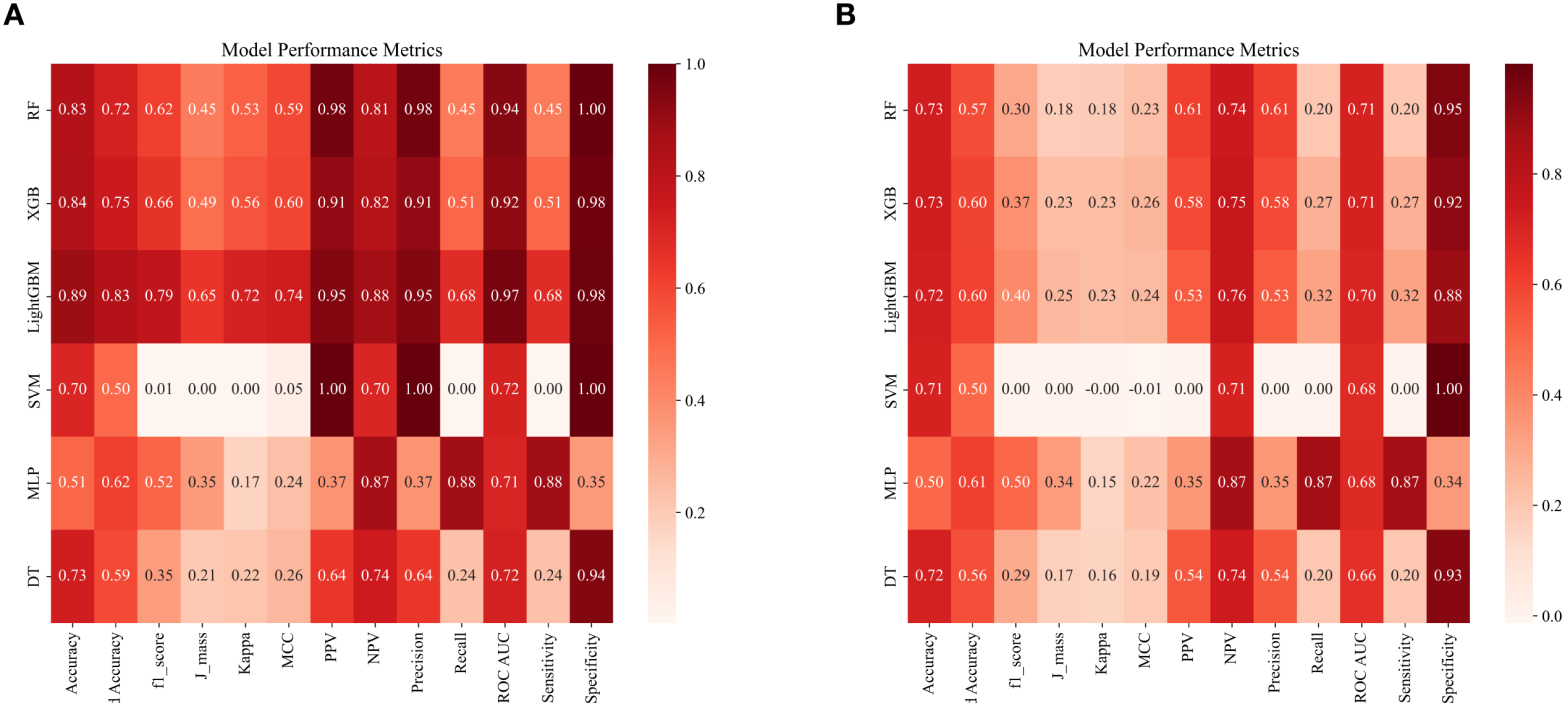
Presents the performance comparison of different machine learning models on the training set **(A)** and the test set **(B)** based on multiple evaluation metrics. The heatmaps show the performance of the Random Forest (RF), LightGBM, Support Vector Machine (SVM), eXtreme Gradient Boosting (XGBoost), Multi - Layer Perceptron (MLP), and Decision Tree (DT) models. Each cell corresponds to the value of a specific evaluation metric, including Accuracy, Balanced Accuracy, F1 score, J - index, kappa, Matthew’s Correlation Coefficient (MCC), Positive Predictive Value (PPV), Negative Predictive Value (NPV), Precision, Recall, Receiver Operating Characteristic Area Under the Curve (ROC AUC), sensitivity (sens), and specificity (spec). A redder color indicates a higher metric value, while a greener color indicates a lower value, so as to demonstrate the effectiveness of the models in both the training and test stages.

Decision curve analysis (DCA) demonstrated that the model constructed using the XGBoost algorithm yielded a higher net benefit compared to both the “universal intervention” and “no-intervention” strategies across the entire range of threshold probabilities, with consistent results observed in both the training cohort (as shown in **Figure 2C**) and the validation cohort (as shown in **Figure 2D**). A comprehensive evaluation of multiple model performance metrics revealed that the XGBoost model exhibited the optimal performance. Notably, the predictive contribution of the number of pregnancies to vitamin D insufficiency in this model was consistent with the negative association observed in Section 3.2, further supporting the associative pattern between the number of pregnancies and serum 25(OH)D levels. To address potential biases arising from imbalanced data distribution, a series of statistical metrics (including Accuracy (ACC), Positive Predictive Value (PPV), Negative Predictive Value (NPV), Sensitivity (SEN), Specificity (SPE), F1-score, and Matthews Correlation Coefficient (MCC)) were calculated to comprehensively assess the predictive capability of the models, and all metrics showed excellent outcomes (as illustrated in **Figure 4**).

In the training set, the Kappa values for the Random Forest (RF), XGBoost, Light Gradient Boosting Machine (LightGBM), Support Vector Machine (SVM), Multilayer Perceptron (MLP), and Decision Tree models were 0.53, 0.56, 0.72, 0.00, 0.17, and 0.22, respectively. As illustrated in **Figure 4**, the corresponding Kappa values in the validation set were 0.18, 0.23, 0.23, 0.00, 0.15, and 0.16. Among these, the XGBoost model showed relatively good stability in both the training and validation sets, and its capture of features such as the number of pregnancies was consistent with the results of the core association analysis.

To interpret the results of the XGBoost model, we plotted SHAP (Shapley Additive Explanations) value graphs. In this visualization, each point corresponds to a participant under a specific feature, and the position of the point on the X-axis (i.e., the actual SHAP value) indicates the impact of that feature on the model’s output for that participant. The results showed that the features in the model, ranked by importance, are as follows: age, body mass index (BMI), blood urea nitrogen, glycated hemoglobin, alkaline phosphatase, alanine aminotransferase, age at first live birth, cholesterol, triglycerides, number of pregnancies, uric acid, creatinine, and aspartate aminotransferase. Among these, the number of pregnancies, as a feature related to the primary research objective, has a SHAP value direction consistent with the negative association identified in Section 3.2, further validating the relationship between the two.

### 3.4 Visualization of feature importance

Using the data presented in **Figures 3A, B**, we conducted SHAP analysis to assess the importance of each feature variable in the XGBoost model and its impact on the model’s predictions. The generated charts consistently show that age, with the highest SHAP value, is the most significant variable among all included factors and a key risk factor for low serum vitamin D. After adjusting for age and BMI, blood urea nitrogen emerges as the second most prominent biochemical predictor and the third most important variable overall. The significance of the remaining variables decreases in sequential order.

## 4 Discussion

The present study was primarily designed to investigate the association between the number of pregnancies and serum 25(OH)D levels among adult females in the US who have had a prior pregnancy. A secondary objective was to develop a machine learning model for predicting vitamin D insufficiency and identifying key influencing factors, with a focus on validating the role of pregnancy count in this context.

Our primary findings confirm a significant and robust negative association between the number of pregnancies and serum 25(OH)D levels: after adjusting for confounding variables, each additional pregnancy was associated with a 0.6 nmol/L decrease in 25(OH)D.

As a secondary analysis, machine learning models—particularly the optimal XGBoost algorithm—further supported this primary association. The XGBoost model identified the number of pregnancies as a non-negligible predictive feature, with its SHAP value direction aligning with the observed negative correlation, thereby validating the primary finding. While the model demonstrated excellent performance in predicting vitamin D insufficiency (AUC = 0.92 in training, stable in validation) and identified age, BMI, and blood urea nitrogen as the most influential features, the retention of pregnancy counts as a relevant predictor reinforces its role in vitamin D homeostasis.

Collectively, these results highlight that cumulative pregnancy history is associated with lower serum 25(OH)D levels, and machine learning models effectively capture this relationship—serving as a complementary tool to validate and extend the primary finding.

With advancements in medical standards and living conditions, public health awareness has heightened, and there is growing focus on the pregnancy period—particularly on the role of vitamin D during pregnancy in shaping maternal and fetal outcomes. For example, research has shown that among Black women, after adjusting for factors such as interleukin-6 (IL-6), the severity of low serum vitamin D correlates with an increased risk of preterm birth. This suggests that achieving adequate vitamin D levels in early pregnancy may be critical for preventing preterm birth (17).

Other research has found connections between newborn vitamin D levels and maternal obesity, maternal age, birth season, preeclampsia, fetal growth restriction, neonatal infection, and fetal distress. Specifically, infants born to mothers with a body mass index (BMI) exceeding 30 kg/m² or born in winter show significantly lower 25(OH)D levels compared to both term and very preterm newborns (19). Low serum vitamin D in neonates can cause serious adverse effects, posing significant risks to their development, though external 25(OH)D deficiency does not affect neonatal anthropometric measurements (20). Additionally, maternal serum 25(OH)D levels may act as an early biomarker for increased fetal adiposity in the third trimester, particularly in pregnancies complicated by diabetes (21). According to previous studies, maternal blood vitamin D is a crucial biomarker with wide-ranging implications for both maternal and fetal health. However, current academic research mainly focuses on the role of vitamin D during individual pregnancies, studying its impact on maternal wellbeing and fetal development in isolation. In contrast, there is a significant gap in research investigating the cumulative effect of multiple pregnancies on maternal vitamin D status. This study addresses this understudied area, contributing to filling the existing void in the literature. The results show that maternal vitamin D levels serve as a critical indicator for the health of both mothers and fetuses. Nevertheless, most existing research has concentrated on the impact of vitamin D during individual pregnancies, with little attention paid to how the cumulative number of pregnancies affects maternal vitamin D levels over time. Studies have shown that insufficient vitamin D levels in pregnant women are negatively correlated with their fasting blood glucose and blood lipid levels, as well as with the level of the immune marker IFN-γ (22). Studies have identified a strong association between vitamin D deficiency and an increased risk of gestational diabetes mellitus, particularly among South Asian women (23). By investigating this understudied relationship, the current study makes a meaningful contribution to addressing this gap, enhancing our understanding of the long-term interplay between maternal vitamin D levels and reproductive history.

In both the training and validation groups, ROC curve results showed that the XGBoost-based method performed best in terms of discriminative performance. This was confirmed by other supplementary evaluation indicators including Accuracy (ACC), Positive Predictive Value (PPV), Negative Predictive Value (NPV), Sensitivity (SEN), Specificity (SPE), F1-score, and Matthews Correlation Coefficient (MCC). According to DCA curve data, all models had some clinical value within a specific threshold range. Specifically, across all thresholds, The XGBoost algorithm generated a predictive model that demonstrated superior clinical utility, as evidenced by a substantial net benefit gain over both the indiscriminate treatment and no-treatment approaches.

We additionally used SHAP values to interpret the XGBoost model, with a focus on validating the role of pregnancy count—our primary variable of interest. Among the included variables, age, BMI, and blood urea nitrogen emerged as the top three significant features, but critically, the number of pregnancies retained meaningful predictive relevance. Notably, the direction of SHAP values for pregnancy count aligned with our primary finding: higher pregnancy numbers were associated with a greater likelihood of low serum 25(OH)D, directly reinforcing the negative association observed in the core analysis. This confirms that the model not only captures broader determinants of vitamin D status but also specifically validates the key relationship central to our study.

To translate our primary finding (the negative association between pregnancy count and 25(OH)D) into practical application, we developed a web tool based on the XGBoost model. Unlike existing tools that focus on demographics or lifestyle (23, 24), our tool integrates reproductive history—specifically the number of pregnancies—as a key input, directly leveraging our core discovery.

This design allows for targeted screening of women with multiple pregnancies, a high-risk group identified in our primary analysis. While similar to other tools in providing user-friendly preliminary evaluation (24), our calculator’s uniqueness lies in its emphasis on pregnancy history, ensuring it addresses the unmet need of identifying individuals whose vitamin D status may be impacted by cumulative reproductive events. By simplifying input of basic biochemical indicators and reproductive information, the tool facilitates efficient detection of at-risk groups, particularly those with multiple pregnancies, thereby supporting targeted interventions aligned with our main findings.

The study population was recruited from American communities, where the prevalence of insufficient 25(OH)D status was 28.9% (23). This aligns with the global landscape: insufficient 25(OH)D status constitutes a critical public health concern worldwide, with regional prevalence rates of 34.22% in Africa, 34.76% in South America (23), and 57.69% in Asia—underscoring the urgency of identifying high-risk populations. For women, existing research has predominantly focused on vitamin D’s role during individual pregnancies, while overlooking the impact of cumulative reproductive history. Our core finding—the negative association between the number of pregnancies and 25(OH)D levels—directly addresses this gap, thereby highlighting the practical significance of developing predictive tools that integrate reproductive factors.

In the field of predictive modeling, existing tools have been developed based on demographic characteristics [e.g., ethnicity, age (24)], lifestyle factors [e.g., outdoor activity duration (25)], or dietary habits [e.g., dairy intake (26)], none of which incorporate reproductive variables such as pregnancy count. In contrast, the key innovation of our tool lies in its integration of gravidity and age at first birth, grounded in our primary finding that multiple pregnancies correlate with lower 25(OH)D levels. This design enables precise identification of women at risk due to cumulative reproductive history, facilitating targeted supplementation strategies.

Furthermore, the large sample size of this study enhances the robustness of our findings. The integration of reproductive variables with biochemical indicators not only distinguishes our tool from existing models but also positions it as a practical vehicle for validating and applying our core discovery. This dual role strengthens the clinical relevance of the tool while reinforcing the importance of pregnancy count in vitamin D homeostasis.

A key strength of this research lies in its large sample size, which enhances the accuracy and reliability of the results. This tool enables efficient and cost-effective screening for insufficient 25(OH)D status through basic biochemical tests and questionnaires, thereby reducing unnecessary public health spending on vitamin D testing. Additionally, it provides an intuitive and robust scientific foundation for health education and further targeted testing.

In addition to using the online calculator data, healthcare providers can offer patients appropriate clinical advice. It facilitates timely intervention for those with a pregnancy history who are at risk of insufficient 25(OH)D status, helping to prevent the development of vitamin D-related diseases and improve population health overall. Primary care providers can assess the potential risk of vitamin D deficiency in individuals using an online tool, based on several easily accessible basic demographic and health information, such as BMI, age, ethnicity, and gender (24).

Nevertheless, This research is not without its limitations. As a cross-sectional study, our design doesn’t have the ability to verify whether more pregnancies directly lower vitamin D levels or if confounding variables (e.g., post-pregnancy dietary changes) drive both; a prospective cohort tracking women through successive pregnancies would clarify this directionality. Hence, prospective investigations in the days ahead will be necessary to validate this correlation. Although we controlled for several confounding variables, unmeasured confounders may still affect the results. The study population was recruited from American communities. Thus, the extent to which these results can be extrapolated to distinct demographic groups demands additional scrutiny.

In conclusion, the core finding of this study is a significant negative association between the number of pregnancies and serum 25(OH)D levels. Based on this primary conclusion, we developed a robust machine learning-based prediction model. The predictive tool derived therefrom, whose core value lies in translating the discovery of “multiple pregnancies correlating with reduced vitamin D levels” into practice, can specifically identify women with multiple pregnancies as a high-risk group. This, in turn, enhances the efficiency of public health management in the field of vitamin D-related health and facilitates the early detection and prevention of 25(OH)D insufficiency.

## 5 Conclusion

Primary finding: Notably, this study focuses exclusively on women with a prior pregnancy, which implies that it is not applicable to women with no pregnancy history. A robust, consistent negative association was identified between the number of pregnancies and serum 25(OH)D levels in U.S. adult women with a prior pregnancy—the target population of this study. This relationship remained significant after adjusting for potential confounders and was stable across diverse subgroups of this population, encompassing varying ages, racial/ethnic backgrounds, and women with diabetes. These results support the hypothesis that multiple pregnancies may exert a cumulative adverse impact on vitamin D status, underscoring reproductive history as a key, yet underappreciated, determinant of long-term vitamin D homeostasis in women with a prior pregnancy.

Secondary outcome: The developed XGBoost model exhibited strong performance in identifying U.S. adult women with a prior pregnancy who have low serum vitamin D. By integrating reproductive factors (notably, the number of pregnancies) with clinical and biochemical indicators, it demonstrated robust predictive efficacy in both training and validation cohorts derived from this population. Critically, its ability to prioritize U.S. adult women with a prior pregnancy and multiple pregnancies as a high-risk group translates the core association into a pragmatic tool for targeted screening, facilitating prioritized interventions for this vulnerable subgroup.

Limitations: The cross-sectional design precludes definitive causal inference; we cannot conclusively attribute lower vitamin D levels to higher pregnancy counts in U.S. adult women with a prior pregnancy, as unmeasured factors (e.g., post-pregnancy dietary patterns, sun exposure) may act as confounding variables. Additionally, the generalizability of the findings and the model beyond U.S. populations—particularly to women with a prior pregnancy in other global regions (e.g., Asia, Africa)—requires validation, given that regional variations in sunlight exposure, dietary patterns, and cultural practices may modulate the pregnancy-vitamin D relationship among non-U.S. women with a prior pregnancy. It is further noted that the present study focused on women with one or more pregnancies, thus excluding women with no pregnancy history (women with zero pregnancies). Future studies encompassing women with zero or more pregnancies (i.e., all women of reproductive age and beyond, regardless of pregnancy history) would be valuable for readers. This broader scope could clarify whether the association between pregnancy and vitamin D differs between women with no pregnancy history and women with one or more pregnancies, while also enhancing the comprehensiveness of insights into vitamin D homeostasis across the full spectrum of female reproductive backgrounds.

Implications: These findings can inform the design of targeted vitamin D supplementation and screening strategies for U.S. adult women with a prior pregnancy and multiple pregnancies, with the potential to enhance maternal and fetal health outcomes in this population on a broader scale. Further longitudinal research focusing on U.S. adult women with a prior pregnancy and regional validation of the predictive model in women with a prior pregnancy worldwide will strengthen the translational impact of these insights, which may also inform tailored interventions for women with a prior pregnancy beyond the U.S. context.

## Data Availability

The raw data supporting the conclusions of this article will be made available by the authors, without undue reservation.
